# Food group intake patterns and nutrient intake vary across low-income Hispanic and African American preschool children in Atlanta: a cross sectional study

**DOI:** 10.1186/1475-2891-11-62

**Published:** 2012-08-29

**Authors:** Deborah Salvo, Jennifer K Frediani, Thomas R Ziegler, Conrad R Cole

**Affiliations:** 1 Current address: Graduate Division of Biological and Biomedical Sciences, Emory University, 1462 Clifton Road, Suite 314, Atlanta, 30322, Georgia, USA; 2 ACTSI, General Clinical Research Center, Emory University Hospital, 1364 Clifton Road, Suite GG-23, Atlanta, 30322, GA, USA; 3 Division of Endocrinology, Metabolism and Lipids, Department of Medicine, Emory University, 1364 Clifton Road, Atlanta, 30322, GA, USA; 4 Current address: Division of Gastroenterology, Hepatology and Nutrition, Cincinnati Children’s Hospital Medical Center, College of Medicine, University of Cincinnati, 3333 Burnett Avenue, Cincinnati 45228OH, USA

**Keywords:** Food group intake patterns, Obesity, Food group analysis, Minority children

## Abstract

**Background:**

The food group intake patterns of low income Hispanic and African American preschool children are not well documented. The aim of this study was to perform a food group intake analysis of low income minority preschool children and evaluate how macronutrient and micronutrient intake compares to Dietary Reference Intakes (DRI).

**Methods:**

A cross sectional study design using three-day food diaries analyzed by dietary analysis software (Nutrient Database System for Research) was used. Children were recruited from well-child clinics at Children’s Healthcare of Atlanta at Hughes Spalding and North Dekalb Grady Satellite Clinic, Atlanta, GA. Low-income, African American and Hispanic preschool age children (n = 291) were enrolled. A total of 105 completed and returned the 3-day food diaries. Chi-squared tests were used to assess demographic variables. The mean percentage of intake per day of specific food groups and sub-groups were obtained (servings of given food group/total daily servings). Food intake data and proportion of children meeting DRIs for macro- and micronutrients were stratified by race/ethnicity, nutritional status, and caloric intake, and were compared using t-tests. Regression models controlling for age, BMI and sex were obtained to assess the effect of total caloric intake upon the proportional intake of each studied food group.

**Results:**

The mean age of African American children was 2.24 ± 1.07 years and Hispanic children 2.84 ± 1.12 years. African Americans consumed more kcal/kg/day than Hispanics (124.7 ± 51 vs. 96.9 ± 33, p < 0.05). Hispanics consumed more fruits (22.0 ± 10.7% vs. 14.7 ± 13.7%, p < 0.05), while African Americans consumed more grains (25.7 ± 7.8% vs. 18.1 ± 6.4%, p < 0.05), meats (20.7 ± 9.0% vs. 15.4 ± 6.1%, p < 0.05), fats (9.8 ± 5.4% vs. 7.0 ± 5.8%, p < 0.05), sweet drinks (58.7 ± 17.1% vs. 41.3 ± 14.8%, p < 0.05) and low-fat dairy products (39.5 ± 19.3% vs. 28.9 ± 12.6%, p < 0.05). Among Hispanics, the proportional intake of fruits, fats and grains varied by total caloric intake, while no difference by total caloric intake was found for the dietary patterns of African Americans. Micronutrient intake also differed significantly between African American and Hispanic children.

**Conclusions:**

Food group intake patterns among low-income children differ by ethnic group. There is a need for more research to guide program design and target nutritional interventions for this population.

## Background

The importance of evaluating food group intake patterns beyond single nutrient data to understand the relationship between food intake and risk of diseases is now increasingly recognized. Studies have documented an association between dietary patterns and cardiovascular disease, Type 2 diabetes, obesity and cancers [1-4]. However, some populations with significant risk factors for chronic diseases have not had their intake patterns extensively evaluated to identify potential risk factors associated with developing these chronic diseases.

It is known that food preferences develop in childhood and are linked to consumption habits in adulthood [5]. The food group intake patterns of children aged 1 through 5 (preschool children) have not been extensively evaluated in the United States, while numerous studies have been conducted for school age children and adolescents [5-8]. Although recommendations addressing feeding patterns for children over the age of two years exist, there are no specific recommendations regarding food groups for children less than two years of age, with very little guidance available on the introduction of complementary foods [6].

The food group intake patterns of preschool minority (Hispanic and African-American) children from low-income families have not been extensively assessed. Hispanics and African Americans represent the groups with the highest rates of Type 2 diabetes mellitus, obesity and cardiovascular disease in the United States [1,9]. Due to the unique socio-cultural elements of these communities, it is possible that distinct patterns of nutrition exist in each group, which could in turn affect their nutritional and health status.

It is important to understand the food group intake of low-income, minority, preschool children to better guide future recommendations, dietary intervention programs and health-related policies directed at improving their nutritional and health status. The objective of our study was to perform a food group-based descriptive analysis of the diets of children aged 1 to 5 years from low-income, minority populations living in the metropolitan area of Atlanta, Georgia, US, and to explore to what extent these individuals meet the DRIs for macronutrients and specific micronutrients.

## Methods

### Study population

This is a secondary analysis of data collected in low-income, minority, preschool children between the ages of 1 and 5 years enrolled in larger study (n = 291) between February 2006 and July 2007 [9,10]. A convenience sampling method was used to recruit low-income, minority children attending well-child clinics at the Children’s Healthcare of Atlanta at Hughes Spalding and North Dekalb Grady Satellite Clinic. The study was explained in either English or Spanish where appropriate. These sites were included because they generally serve low-income families that may benefit from Women Infant and Children supplemental nutrition program (WIC) or Supplemental Nutrition Assistance Program (SNAP). During study enrollment, data was collected on, ‘enrollment on WIC status, mother’s educational level and type of insurance’, which were used as surrogate markers for socio-economic status [9]. Children with a history of sickle cell anemia or acute diarrheal or respiratory infections were excluded. Information is not known on those individuals who chose not to participate in the study.

Informed consent was obtained in English or Spanish from the primary caregivers and the study was approved by the Research Oversight Committee of Grady Memorial Hospital and the institutional review boards (IRB) of Emory University and Children’s Healthcare of Atlanta. The race/ethnicity of the child was self-identified by the caregiver who gave consent for participation in the study.

### Food records

Dietary information was assessed using food diaries in English or Spanish collected over three consecutive days for each participant. Each participant was given detailed instruction as well as measuring cups, spoons and a picture guide of portion sizes to complete the diaries. They were also given instructions to provide details on brand names and food preparation methods. Caregivers were also instructed to provide information only on what the child ate, and not what he or she may have been served. Of the initial 291 children enrolled, 182 were excluded from the present analysis due to lack of any food diaries, while three were excluded because of insufficient food diaries (< 3 days), and one participant that was determined to be an outlier (total caloric intake = 15.4 kcals/kg/day) was also excluded. The final sample size for this analysis was 105 children (Hispanic, n = 42; Black, n = 63). The majority (n = 103, 98.1%) of primary caregivers were biological mothers, while the other two were legal guardians. The instructors were dietitians and were assisted by Spanish language interpreters. The diaries were checked when received by the research dietitian and research coordinator. Diaries that were not adequately completed or did not include all three days were excluded. Patients with intake considered outside feasible levels were excluded. Foods were analyzed for calories, protein, carbohydrates, calcium, iron, zinc, vitamin D and food groups determined by a research dietitian (JKF) using the Nutrient Database System for Research (NDSR; University of Minnesota, MN).

The present study was limited to participants who completed three-day food records. It has been reported that this method provides a reliable and valid estimation of intake for this age group [11].

### Descriptive analysis by food group

Total calories and food group servings per day were calculated for each participant. The food groups included: fruits, vegetables, grains, meats, nuts and seeds, dairy products, fats and oils, sweets and miscellaneous products. Mean percentage of intake per day were obtained per food group. These percentages of intake were calculated using the number of food group servings divided by the total daily serving intake from all food groups per participant.

Analysis of food sub-groups was done to further examine choices within some food groups. Comparisons were done for the proportional intake of the food sub-groups over total dietary intake, and over the food group from which the sub-groups were derived (e.g. for fruits, the proportion of fruit intake that came from whole fruits was examined, as well as examining the proportion of whole fruits over total daily dietary intake of all foods). The sub-group analysis included studying the proportion of fruits and vegetable intake derived only from whole fruits, the proportion of low- or non-fat products within the dairy category, the proportion of healthier fats and oils, and the proportion of sweet intake derived from sweetened beverages.

Data analysis included stratification by sex, age and race/ethnicity. The stratification by ethnicity allowed for comparison of dietary patterns across Hispanic and African American preschool children. Further stratification by total caloric intake was done to compare the proportional intake per food group among children with high caloric intake versus children with low caloric intake. High total caloric intake was defined as being above the DRI (Estimated Energy Requirement, EER) for the given age and sex groups, while low caloric intake was defined as being lower or equal to the EER [12]. Mean comparisons (t-tests) and linear regression models (all dependent variables were assessed for normality) were built, and significance was assessed at the 0.05 and 0.10 levels respectively.

### DRI compliance

A complementary analysis to explore the extent to which the participants met the age-related DRI recommendations for macronutrients and selected micronutrients was performed. The cutoffs used for adequate intake correspond to the parameters described in the U.S. Dietary Reference Intakes for Recommended Dietary Allowances (RDA): 13 g/protein/d for children ages 1 to 3 years, and 19 g/protein/d for children ages 4 to 5 years; 130 g/day of carbohydrates for ages 1–5; 500 mg/d of calcium for ages 1 to 3 years, and 800 mg/d for those above 4 years; 7 mg/d and 10 mg/d of iron for children ages 1 to 3 years and ≥ 4 years, respectively; 3 mg/d of zinc for children ages 1 to 3 years, and 5 mg/d for ≥ 4 years; and 600 IU (15 mcg) of vitamin D/d [12,13]. These micronutrients were selected for analysis because of their biologic significance and relatively high deficiency rates reported in low-income children within the US population [9,14-16].

The second phase of this analysis examined the compliance towards recommendations concerning the distribution of macronutrients in the diet. The DRIs (RDA) establish that children aged 1 to 3 years should have a diet with the following distribution: 5–20% protein, 45–65% carbohydrates and 30–40% fat. For children over the age of 4 years, the recommended macronutrient distribution is 10–30% protein, 45–65% carbohydrates and 25–35% fat [13].

All of the above analyses were stratifying by sex, ethnicity and weight status (obese or non-obese). Weight and height were measured as previously described [9] and weight status was determined using CDC growth charts [17]. For children < 2 years of age, weight for height above the 95th was considered “obese”, and for those 2–5 years, CDC charts for BMI (kg/m^-2^) were used, with the 95th percentage as the cut-point for obesity [17]. Chi-squared tests were performed.

All statistical analyses were performed using SAS 9.3 (SAS Institute Inc., Cary, NC, USA).

## Results

The demographic characteristics of the full sample recruited (n = 291), the children who returned both two or three days of food diaries (n = 109) and the final 105 children included in the analysis (completed 3 days of food diaries) are shown in Table 1. There was no difference in sex, age, and prevalence of obesity between the final sample used for this analysis and the entire sample recruited. The prevalence of obesity was significantly higher in the Hispanic children compared to the African American children, (21.3% vs. 14%; p < 0.05) for the full sample (n = 291) recruited. However, there was no difference in the prevalence of obesity in the final sample used for analysis (Hispanic 21.4% vs. African American11.1%, p = 0.1) due to the smaller sample size.

**Table 1:** Demographic characteristics of the full sample versus participants with 2 and 3 food diaries

	**Full sample**			**Participants with at least 2 food fiaries**			**Participants with 3 food fiaries**		
	**Hispanic**	**African American**	**Total**	**Hispanic**	**African American**	**Total**	**Hispanic**	**African American**	Total
Male	60* (42.6%)	85 (56.7%)	145 (49.8%)	17* (39.5%)	39 (59.1%)	56 (51.4%)	16* (38.1%)	38 (60.3%)	54 (51.4%)
< 3 years	78* (55.3%)	115 (76.7%)	193 (67.4%)	22* (51.2%)	51 (77.3%)	76 (67.0%)	21* (50%)	48 (76.2%)	69 (65.7%)
Obese	30* (21.3%)	21 (14.0%)	51 (17.5%)	9 (20.9%)	8 (12.1%)	17 (15.6%)	9 (21.4%)	7 (11.1%)	16 (15.2%)
WIC Enrolled	103 (73.0%)	106 (70.7%)	209 (71.8%)	32 (75.0%)	47 (71.2%)	79 (72.5%)	32 (76.2%)	45 (71.4%)	77 (73.3%)
High caloric intake	NA	NA	NA	20* (46.51%)	48 (72.7%)	68 (62.4%)	20* (47.6%)	46 (73.0%)	66 (62.9%)
TOTAL	141 (48.1%)	150 (51.4%)	291 (100%)	43 (39.4%)	66 (60.6 %)	109 (100%)	42 (40.0%)	63 (60.0%)	105 (100%)

*Difference across ethnicities p < 0.05.

High caloric intake was defined as being higher than the DRI (EER) for the specific age and sex.

NOTE: No significant differences were found among any of the categories when comparing the samples of 2 food diaries and 3 food diaries to the full sample.

Among the 105 children included in the final group with completed food records returned, the mean age was 2.5 ± 1.3 years; with 16 (15.2%) children characterized as obese. The daily caloric intake ranged from 42.7 to 272.9 kcal/kg/day (mean 112.7 ± 43.3 kcal/kg/day), while the number of total servings ranged from 9.2 to 33.2, with a mean of 17.52 ± 9.4, and no difference by race/ethnicity. African American children consumed significantly more kcal/kg/day than the Hispanic children (124.7 ± 49.5 kcal/kg/day vs. 96.9 ± 32.8 kcal/kg/day, p < 0.05), and more African American children had a high total daily caloric intake (73.0% vs. Hispanic 47.6%, p < 0.05). Overall, obese children consumed significantly higher kcal/kg/day than non-obese children (obese, 116.1 ± 47.2 vs. 92.1 ± 33.4 kcal/kg/day, p < 0.05). However, when stratified by ethnicity, only the obese African American children consumed more daily calories than their non-obese counterparts (127.1 ± 52.4 vs. 91.1 ± 22.9 kcal/kg/day, p < 0.05).

### Proportional intake by food group

The mean percentage intake (obtained using food group servings divided by total daily servings per participant) for each of the evaluated food groups showed that for the lowest quartile (bottom 25%) of children in the study, fruit intake represented 9.8%, and vegetables 5.0% of the daily intake. Furthermore, for the lower quartile, meat intake represented 12.5% of the daily intake, and increases to 23.2% for the top quartile.

When stratified by race/ethnicity (Table 2), Hispanics consumed a higher proportion of fruits in their diet compared to African Americans (22.0 ± 10.7% vs. 14.7 ± 13.7%, p < 0.05). However, Hispanic children consumed a lower proportion of grains compared to the African American children (18.1 ± 6.4% vs. 25.7 ± 7.8%, p ≤ 0.05). African Americans had a higher intake of meats (20.7 ± 9.0% vs. 15.4 ± 6.1%, p < 0.05) than Hispanics. There was no difference between African Americans and Hispanics in their intake of sweets, nuts, seeds, vegetables and miscellaneous foods. When stratified by sex, the food group intake patterns identified for fruits, grains, meats and dairy products among Hispanics and African Americans was unchanged. However, African American boys consumed more fat than Hispanic boys (10.8 ± 5.4% vs. 6.9 ± 4.8% respectively, p < 0.05) with no difference in the fat intake between African American and Hispanic girls. There was no difference when stratified by age (data not shown).

**Table 2:** Distribution of intake within food groups and sub-categories of food groups stratified by race/ethnicity

		**Hispanic**		**African-American**		
**Food group (Food sub-group)**	**Food group as % of total servings per day**	**Sub-group item as % of total servings per day**	**Sub-group item as % of food group**	**Food group as % of total servings per day**	**Sub-group item as % of total servings per day**	**Sub-group item as % of food group**
Fruits (All but juices)	22.0 ± 10.7*	10.3 ± 6.0*	50.0 ± 26.8*	14.7 ± 13.7	4.1 ± 5.1	36.4 ± 36.9
Vegetables (All but juices)	9.4 ± 5.0	9.3 ± 5.0	99.7 ± 1.8	9.5 ± 5.2	9.4 ± 5.3	98.4 ± 7.3
Dairy (Low/Non Fat)	16.6 ± 8.4*	4.1 ± 2.8	28.9 ± 12.6*	9.3 ± 6.4	3.3 ± 2.1	39.5 ± 19.3
Fats and Oils (Low Fat)	7.0 ± 5.8*	0.0 ± 0.0	0.0 ± 0.0*	9.8 ± 5.4	0.6 ± 0.2	6.1 ± 1.8
Sweets (Sweet Drinks)	8.4 ± 2.4	2.6 ± 1.7	41.3 ± 14.8*	6.5 ± 2.1	4.4 ± 2.4	58.7 ± 17.1
Grains	18.1 ± 6.4*	*NA*	*NA*	25.7 ± 7.8	*NA*	*NA*
Meats	15.4 ± 6.1*	*NA*	*NA*	20.7 ± 9.0	*NA*	*NA*
Nuts and Seeds	0.3 ± 0.8	*NA*	*NA*	0.8 ± 2.6	*NA*	*NA*
Miscellaneous	0.03 ± 0.03	*NA*	*NA*	0.03 ± 0.03	*NA*	*NA*

*difference across ethnicities p < 0.05.

Sub-group analysis (Table 2) revealed that whole fruit intake was higher among Hispanic compared to African American children, (10.3 ± 6.0% vs. 4.1 ± 5.1%, p < 0.05). A higher proportion of dairy products consumed were low-fat or non-fat among African American children compared to that consumed by Hispanic children (39.5 ± 19.3% vs. 28.9 ± 12.6%, p < 0.05). Although the intake of healthier fats and oils was significantly higher among African American children (p < 0.05), the intake of this food sub-group among both races was extremely limited, being zero for the Hispanic children and 0.6 ± 0.2% of the total food intake for African Americans. African American and Hispanic children were found to have a very high consumption of sweet drinks, yet African Americans had a proportionally higher intake (58.7 ± 17.1% vs. 41.3 ± 14.8%, p < 0.05).

Among African Americans, no difference was found for the mean proportional intake of any of the food groups studied when stratifying by total daily caloric intake (Table 3). Hispanics in the low total caloric daily intake group consume significantly more fruits, and less grains and fats/oils than those in the high intake group. Marginal significance values (p < 0.10) show that among Hispanics, there may be a higher intake of vegetables and lower intake of sweets among the low total daily caloric intake group. These associations remain consistent not only for the comparison of means of proportional intake per food group, but also for the regression models that control for the effect of age, sex and BMI.

**Table 3:** Intake per food group across ethnicities, stratified by total caloric intake: Mean proportional intakes and regression models

	**Mean proportional intake (% over total servings per day)**		**Effect of total caloric intake on mean proportional intake by food group**
	**Low caloric intake**	**High caloric intake**	**(linear regression)**
Fruits	20.00 ± 11.93 *	15.03 ± 9.86	–4.79 ± 2.26 *
Hispanic	24.37 ± 12.34 *	18.18 ± 5.96	–5.63 ± 2.04 **
African American	16.46 ± 10.70	13.53 ± 10.12	–2.14 ± 3.04
Vegetables	9.74 ± 5.67	9.30 ± 4.80	−0.49 ± 1.07
Hispanic	10.37 ± 5.51**	7.72 ± 2.64	−2.89 ± 2.02 **
African American	10.15 ± 6.05	9.11 ± 4.62	−0.33 ± 1.03
Grains	19.12 ± 8.26 *	24.80 ± 7.40	6.17 ± 1.61 *
Hispanic	16.55 ± 6.99 *	20.68 ± 4.52	5.11 ± 1.94 *
African American	24.6 ± 8.26	26.49 ± 7.51	3.22 ± 2.20
Meats	17.13 ± 9.30	19.48 ± 7.68	2.21 ± 1.74
Hispanic	15.25 ± 6.52	15.72 ± 5.63	−0.70 ± 1.01
African American	19.69 ± 10.45	21.45 ± 7.86	1.75 ± 2.63
Dairy	15.43 ± 10.18 *	10.37 ± 5.07	−5.60 ± 1.62 *
Hispanic	17.62 ± 9.58	15.02 ± 6.13	−4.63 ± 2.54 **
African American	10.03 ± 8.11	8.83 ± 5.04	−2.66 ± 1.91
Fats and Oils	7.70 ± 5.75 **	9.91 ± 5.53	2.10 ± 1.66 **
Hispanic	5.41 ± 3.98 *	9.55 ± 7.42	3.41 ± 1.81 **
African American	10.15 ± 6.36	9.60 ± 4.63	−0.73 ± 1.62
Sweets	6.62 ± 5.84	8.02 ± 6.70	1.50 ± 1.28
Hispanic	6.85 ± 4.04 **	10.85 ± 6.58	4.35 ± 2.50 **
African American	6.53 ± 3.60	7.16 ± 5.47	0.35 ± 1.51
Nuts and seeds	0.91 ± 1.82	0.38 ± 0.80	−0.46 ± 0.43
Hispanic	0.36 ± 0.95 **	0.19 ± 0.56	−0.30 ± 0.26
African American	1.04 ± 3.03	0.56 ± 0.78	−1.01 ± 0.75
Miscellaneous	0.03 ± 0.03	0.03 ± 0.03	−0.01 ± 0.01
Hispanic	0.03 ± 0.04	0.02 ± 0.03	−0.02 ± 0.01
African American	0.03 ± 0.03	0.03 ± 0.03	0.001 ± 0.01

- Low caloric intake is group of reference.

- All models are controlled for sex, age and BMI.

- High caloric intake; for males 1 to 3 years: > 1046 kcal/day; for males >3 years: >1742 kcal/day; for females 1 to 3 years: >992 kcal/day; for females > 3 years: > 1642 kcal/day. Cut points based on DRIs (EER).

* Difference between low and high caloric intake p < 0.05.

** Difference between low and high caloric intake p < 0.10 (marginal significance).

### DRI compliance

Unstratified analysis of the diet intake records revealed that all children met the DRI (RDA) for protein, and the majority met the DRI for zinc (94.3%, n = 99). However, a proportion of children consumed less than the recommended levels for carbohydrates 19.0% (n = 20), calcium 35.2% (n = 37), vitamin D 96.2% (n = 101) and iron 25.7% (n = 27). When stratified by race/ethnicity, more Hispanic children met the recommendations for intake of all of these nutrients, compared to African American children, while iron intake was similar between groups (Table 4). The unstratified analysis revealed that among low-income, minority, preschool children, compliance to the suggested distribution for protein was high (89.5%), but low for carbohydrates (59.0%) and fats (39.0%).

**Table 4:** DRI compliance stratified by race/ethnicity

	**Below DRI n (%)**	
**Race/Ethnicity**	**Hispanic**	**African-American**
Protein	0 (0.0%)	0 (0.0%)
Carbohydrates	8 (19.0%)	12 (19.0%)
Calcium	13 (31.0%)	24 (38.1%)
Iron	13 (31.0%)	14 (22.2%)
Zinc	1 (2.4%)	5 (7.9%)
Vitamin D	40 (95.2%)	61 (96.8%)

Percentage of children below the DRI (RDA) for macronutrients and selected micronutrients.

Stratification (Table 5) showed that fewer African American children consumed diets containing recommended protein amounts within the recommended distribution compared to Hispanics (85.7% vs. 97.6%, p < 0.05). For carbohydrate intake, more African Americans were below the recommended distribution than Hispanics (23.8% vs. 7.1%, p < 0.05), while more Hispanics had a fat intake below the recommended distribution than Hispanics (52.4% vs. 27.0%, p < 0.05).

**Table 5:** RDA macronutrient distribution compliance stratified by race/ethnicity and nutritional status

	**Macronutrient distribution**		
	**n (%)**		
	**Protein**	**Carbohydrates**	**Fats**
Stratification by race/ethnicity			
Hispanic			
Within adequate distribution	41 (97.6)*	27 (64.3)	13 (31.0)
Below adequate distribution	0 (0.0)	3 (7.1)*	22 (52.4)*
Above adequate distribution	1 (2.4)	12 (28.6)	7 (16.7)
African American			
Within adequate distribution	54 (85.7)	35 (55.6)	28 (44.4)
Below adequate distribution	2 (3.2)	15 (23.8)	17 (27.0)
Above adequate distribution	7 (11.1)	13 (20.6)	18 (28.6)
Stratification by Weight Status			
Obese			
Within adequate distribution	14 (87.5)	11 (68.8)*	6 (37.5)
Below adequate distribution	0 (0.0)	2 (12.5)	7 (43.8)
Above adequate distribution	2 (12.5)	3 (18.8)	3 (18.8)
Non-Obese			
Within adequate distribution	82 (92.1%)	51 (57.3)	30 (33.7)
Below adequate distribution	1 (1.1)	16 (18.0)	37 (41.6)
Above adequate distribution	6 (6.7)	22 (24.7)	22 (24.7)

* Difference across strata p < 0.05.

Adequate distribution:

1–3 years: 5–20% protein, 45–65% carbohydrates and 30–40% fat.

4–5 years: 10–30% protein, 45–65% carbohydrates and 25–35% fat.

Similarly, stratification by weight status was performed to assess compliance to the DRI for macronutrient distribution (Table 5). Sixteen children (15.2%) were classified as obese, using BMI z-scores. Proportionally, there was a higher compliance to carbohydrate intake among obese children than among non-obese (68.8% vs. 57.3%, p < 0.05). The proportion of obese children with low-fat intake was similar to that of non-obese children with low-fat intake (43.8% vs. 41.6%). There was no difference between the proportion of non-obese children that met the recommended distribution of intake of protein when compared to obese children (92.1 vs. 87.5%). In spite of similar obesity rates, Hispanic children showed higher compliance rates to RDAs for macro- and micronutrients than African Americans.

## Discussion

The objective of this study was to perform a food group-based analysis of low-income, minority, preschool children, rather than studying individual nutrient intake [18-20]. Significant differences were identified between Hispanic and African American children, suggesting influences of social and cultural practices on dietary patterns. Previous studies have used methodologies such as dietary scoring to analyze food patterns among large population groups [2,21,22]. We decided not to adopt a pre-existing scoring mechanism because these were designed and validated only for children over the age of 2 years, while the goal of this study is to evaluate the food patterns of children aged 1 to 5 years of age. This methodology employing servings per food groups as the unit of analysis to assess dietary patterns have been previously reported as a validated method to assess nutrient intake [5,23-25].

Hispanics consumed a much larger proportion of fruits and whole fruits in their diet than African Americans which is consistent with previous studies [26]. However this study is the first to report it for this age group. Culturally, high fruit consumption is common among Hispanics, especially in families that migrate from rural areas, as are the majority of Hispanic migrants residing in urban Georgia [27]. While confirming previous findings, our results also provide further insight on this behavior, showing that not only is total and whole fruit consumption higher than that of African Americans, but juice consumption is also very high in this population, accounting for half of their total fruit intake, and 10.3% of their caloric intake. This, in addition to the high intake of sweet drinks in both Hispanic and African American children, is a relevant finding as investigators recently reported lack of activation of satiety signals in the gut with liquid food as opposed to solids [28,29]. Also fructose overrides the regulatory glycolytic step leading to higher rates of fat production in the liver, increasing abdominal fat deposition [30-32]. Moreover, sweetened beverages, including fruit juices, are associated with negative health outcomes due to increase adiposity gain in low-income children [33]. Carbohydrate malabsorption is also high in infants consuming sorbitol-containing juices with high fructose-to-glucose ratio which has a negative impact on metabolism and activity [34]. The American Academy of Pediatrics [35] recommends reducing overall fruit juice intake among children and replacing it with whole fruits due to these documented negative effects associated with intake of large volumes of fruit juices.

As in previous studies, Hispanics were found to have a significantly higher intake of dairy products than African Americans (16.6 ± 8.4% vs. 9.3 ± 6.4%) [36-40]. Milk and other dairy products constitute a primary source of calcium, especially for children [41-43]. Vitamin D intake for both groups was found to be extremely low, with more than 95% of the studied population not meeting the RDA. Vitamin D status and consumption in this population has been evaluated and extensively discussed [10].

African American children had a higher consumption of meats than Hispanic children which contributed to the higher number of African American children exceeding the recommended distribution for protein (11.1% vs. 2.4% for Hispanics). These findings are consistent with previous knowledge of food habits among African Americans in the US, particularly in the South, where high meat, egg and poultry intake is common [44-47]. The lower intake of meats by Hispanics could contribute to their higher prevalence of low iron intake reported. However, more Hispanics than African Americans met the RDA for zinc (97.6% vs. 92.1%). This is a noteworthy finding as the main dietary source of zinc and iron is meat [9,43]. The other rich sources of zinc consumed by both groups include dairy products and legumes [9,48,49].

Based on these results, we recommend the following strategies to improve the diet quality of low-income, preschool Hispanics: 1) Encourage a high fruit intake among this group, while deemphasizing the need for fruit juices; 2) Maintain current levels of dairy intake, while promoting the use of low-fat dairy products, emphasizing these are safe for healthy children over the age of two years; 3) Increase the intake of iron-rich products such as meat, poultry and legumes. 4) Provide information on the appropriate portion sizes to prevent high calorie diets, which in this population may be also associated to less healthy dietary patterns. 5) Emphasize the use of culturally appropriate preparations to combine cereals and legumes to provide a high quality protein, yet low-fat meal. A good example for this is the combination of corn tortillas with black beans. For preschool, low-income African Americans, our results suggest that: 1) A higher total fruit intake should be promoted, while emphasizing the consumption of whole fruit as opposed to juices; 2) Meat intake should be reduced if the intake of protein is excessive. Replacing some of the animal protein with vegetable protein, which contains other vital nutrients is also recommended; 3) Portion sizes should be reduced globally due to obesity being partly driven by a higher caloric intake in this population. Finally, our data suggests the following strategies should take place for both Hispanics and African Americans: 1) Increase vegetable intake by incorporating one or two vegetable portions at both lunch and dinner; 2) Reduce the consumption of sweets with a strong emphasis on the reduction of sweet beverage intake; 3) Encourage healthy snacking by encouraging more nutrient dense foods and discouraging empty calories. This includes reducing exposure to fast food which has been shown in other studies to be a significant contributor to childhood obesity [50]. For interventions to have a higher probability of success, culturally appropriate strategies should be adopted to improve nutrition behaviors in these groups. The promotion of traditional corn tortillas (high in calcium, fiber and low in calories) among Hispanics rather than flour tortillas (higher in fat and calories) is a good example.

Many of the limitations of this study are due to the sample size and that this is the secondary analysis of existing data. The original sample size calculation was based on identifying differences in serum micronutrient levels and the study was not powered to identify differences between food groups [9,10]. There was also a higher representation of African-American children in the families who completed food diaries. The data collected did not measure or control for physical activity levels, fast-food intake and environmental influences. It has been reported that Hispanic and African-American children have lower levels of physical activity than the rest of the population [51]. Investigators have also observed that obese infants have lower activity levels, compared to non-obese infants and could in turn lead to obese preschoolers [52]. Fast-food is known to be a major contributor to childhood obesity, and its consumption is highly prevalent among all groups of children. It is highest in African Americans, particularly those residing in the South. Children with a high intake of fast-food have been reported to have higher total caloric intake, higher fat, sugar and sodium intake, and lower intake of fruits and vegetables [50]. The effect of contextual factors such as the food environment (higher density of fast-food outlets in low-income neighborhoods) should be addressed in future studies [53,54]. Foreign-born Hispanics are at lower risk for obesity and have healthier dietary patterns than US born Hispanics [55-58]. The level of acculturation has also been found to be an important contributor to obesity independent of duration of residence in the US [56,58-61]. These facts have led to the healthy immigrant effect or the immigrant paradox hypothesis. It highlights the fact that upon arrival, Hispanic immigrants are the healthiest of all groups in the US, and have a diet high in fruits and vegetables and low in fat. After time, presumably due to environmental influences, these Hispanics become the unhealthiest group as they begin to consume a high-fat, high-protein diet, also low in fruits and vegetables, and increase their fast-food intake [62,63]. Studies addressing the influence of diet on obesity in Hispanics should control for these social influence factors. It is also necessary to explore the potential complex gene-environment interaction, which in combination with caloric intake and dietary patterns could have a role in this observed obesity epidemic among minority groups.

## Conclusions

This study reveals that food group and nutrient intake patterns among low-income Hispanics and African Americans between the ages of 1 to 5 years living in Atlanta are different. It provides preliminary data for a larger, more definitive study relevant for program design targeting nutritional interventions tailored for each community. It is important to understand that a shared problem does not always imply a shared solution.

## Abbreviations

RDA : Recommended Dietary Allowance; DRI : Dietary Reference Intakes; EER : Estimated Energy Requirements; BMI : Body Mass Index; CDC : Centers for Disease Control and Prevention; WIC : Women Infant and Children Program; SNAP : Supplemental Nutrition Assistance Program; IRB : Institutional Review Board; NDSR : Nutrient Database System for Research.

## Competing interests

The authors declare no personal or financial competing interests related to this work.

## Authors’ contributions

DS contributed to the conception and design of the study; performed the statistical analyses and interpretation of the data; drafted the manuscript. JKF contributed to the data collection, drafting and critical revision of the manuscript. TRZ contributed to conception and design of the study and the critical revision of the manuscript. CRC contributed to the conception and design of the study, data collection, and interpretation of the data and the critical revision of the manuscript. All authors read and approved the final manuscript.
